# Iron overloaded polarizes macrophage to proinflammation phenotype through ROS/acetyl‐p53 pathway

**DOI:** 10.1002/cam4.1670

**Published:** 2018-07-10

**Authors:** Yun Zhou, Ke‐Ting Que, Zhen Zhang, Zu J. Yi, Ping X. Zhao, Yu You, Jian‐Ping Gong, Zuo‐Jin Liu

**Affiliations:** ^1^ Chongqing Medical University Chongqing China

**Keywords:** acetylated p53, iron, macrophages, p53, reactive oxygen species

## Abstract

**Purpose:**

Macrophages play critical roles in inflammation and wound healing and can be divided into two subtypes: classically activated (M1) and alternatively activated (M2) macrophages. Macrophages also play important roles in regulating iron homeostasis, and intracellular iron accumulation induces M1‐type macrophage polarization which provides a potential approach to tumor immunotherapy through M2 tumor‐associated macrophage repolarization. However, the mechanisms underlying iron‐induced M1 polarization remain unclear.

**Methods:**

Western blotting, qRT‐PCR, and flow cytometry were used to detect the polarization indexes in RAW 264.7 murine macrophages treated with iron, and Western bloting and qRT‐PCR were used to detect p21 expression. The compound 2,7‐dichlorofluorescein diacetate was used to measure reactive oxygen species (ROS) levels in macrophages after iron or *N*‐acetyl‐l‐cysteine (NAC) treatment. The p300/CREB‐binding protein (CBP) inhibitor C646 was used to inhibit p53 acetylation, and Western bloting, qRT‐PCR, and immunofluorescence were used to detect p53 expression and acetylation. BALB/c mice were subcutaneously injected with H22 hepatoma cells, and macrophage polarization status was investigated after tail intravenous injection of iron. Immunohistochemical staining was used to evaluate the protein expression of cluster of differentiation 86 (CD86) and EGF‐like module‐containing mucin‐like hormone receptor‐like 1 (F4/80) in the subcutaneous tumors.

**Results:**

Iron overload induced M1 polarization by increasing ROS production and inducing p53 acetylation in RAW cells, and reduction in ROS levels by NAC repressed M1 polarization and p53 acetylation. Inhibition of acetyl‐p53 by a p300/CBP inhibitor prevented M1 polarization and inhibited p21 expression. These results showed that high ROS levels induced by iron overload polarized macrophages to the M1 subtype by enhancing p300/CBP acetyltransferase activity and promoting p53 acetylation.

## 1 INTRODUCTION

Iron is a vital trace material for cell survival and metabolism and is involved in DNA synthesis, red blood cell production, mitochondria biosynthesis, energy metabolism, and oxygen transport. Dietary iron is mainly absorbed in duodenal mucosa. Iron is oxidized from Fe^2+^ to Fe^3+^ by hephaestin and then released into the bloodstream through ferroportin (FPN). Free iron binds to transferrin and is imported into cells partly through the action of the transferrin receptor (TfR). Iron is stored in intracellular ferritin or a labile iron pool and exported into the circulation by FPN. Intracellular iron concentration is mainly regulated by hepcidin, which is also known as liver‐expressed antimicrobial peptide. Hepcidin can be rapidly synthesized in the liver and is mainly regulated by serum iron level and body iron requirement. When hepcidin level is abnormally low, iron overload occurs due to increased FPN‐mediated iron efflux from storage. Excess serum iron increases the level of hepcidin, thus inducing FPN degradation and reducing iron release into the bloodstream.^1^, ^2^

Aerobic cells can produce reactive oxygen species (ROS), which are mainly produced in the mitochondria, endoplasmic reticulum, plasma membrane, and cytosol under physiological conditions.^3^ Immunologically activated macrophages produce ROS, which are associated with enhanced antimicrobial activity.^4^ ROS mainly include superoxide, hydrogen peroxide, singlet oxygen, ozone, hypohalous acids, and organic peroxides, all of which are byproducts of aerobic metabolism. Levels of ROS are mainly regulated by nonenzymatic antioxidants (eg, α‐tocopherol and glutathione) and antioxidant enzymes (eg, superoxide dismutase and catalase).^5^ ROS physiologically regulates the synthesize of some kinds of proteins, such as phosphatase and tensin homologue, epidermal growth factor receptor, protein kinase B, and histone deacetylases,^6^, ^7^, ^8^, ^9^ and oxidizes DNA to promote transcription.^4^

P53 acts as a tumor suppressor in many tumor types, it can induce growth arrest or apoptosis determined by cellular damages or physiological circumstances. P53 protein binds to DNA‐specific response elements to arrest the cell cycle in G1/S phase and improve genome stability after cell DNA damage, thus activating DNA damage repair system. When DNA is severely damaged and cannot be repaired, p53 protein induces apoptosis and senescence to prevent malignant proliferation of cells.^10^ Acetylation of p53 increases the stability and affects the transcriptional activity of p53 protein. In general, acetylation of p53 is mediated by two different groupings of acetyltransferases, p300/CBP/PCAF or Tip60/MOF/MOZ.^11^

Macrophages are important components of the immune defense and antigen presentation in vivo. They have good plasticities and can be polarized into different subtypes with various stimuli. Polarized macrophages can be divided into two distinct states according to immunological study. Classically activated (M1) macrophages are polarized by lipopolysaccharides and interferon‐γ and highly express tumor necrosis factor alpha (TNF‐α), interleukin 1 (IL‐1), and inducible nitric oxide synthase (iNOS). Alternatively activated (M2) macrophages are polarized by IL‐4 and exhibit high constitutive expression of arginase I, IL‐6, cluster of differentiation 206 (CD206), transforming growth factor beta (TGF‐β), and IL‐10. M1 macrophages exert proinflammatory and antitumor activities, whereas M2 macrophages exhibit immunosuppressive and tumor‐promoting characteristics. Macrophages also play important roles in iron metabolism because of their versatile roles during innate immunity. Some studies have shown that M1 macrophages sequester iron in the cytoplasm and express low levels of TfR and FPN. However, the expression of iron metabolism‐related proteins in M2 macrophages is different than that in M1 macrophages.^1^ Another study^12^, ^13^ showed that increasing levels of intracellular iron induced polarization of proinflammation macrophages. The results of these studies suggest that regulating iron concentration in macrophages may be a novel and effective strategy for cancer immunotherapy. However, the mechanisms underlying iron regulation in polarized macrophages remain unclear. Some studies^14^, ^15^, ^16^, ^17^ have shown that iron can induce high levels of ROS and DNA damage. Alternatively, ROS may also induce p53 due to DNA damage.^3^

## 2 MATERIALS AND METHODS

### 2.1 Materials

Ferric sulfate and ferrous sulfate heptahydrate were purchased from Energy Chemical Technology Co., Ltd. (Shanghai, China). Ferric citrate was purchased from Sigma‐Aldrich (St. Louis, MO). Fetal bovine serum (FBS) was purchased from Invitrogen (Carlsbad, CA). Dulbecco's Modified Eagle's Medium (DMEM) was obtained from Sigma (Yuzhong District, Chongqing, China). Antibodies against p21 and C646, a p300/CREB‐binding protein (CBP) inhibitor, were purchased from Abcam Trading Co. Ltd. (Shanghai, China). *N*‐acetyl‐l‐cysteine (NAC), dihydroethidium (DHE), and a ROS Detection Kit were purchased from Beyotime Company (Jiangsu, China). Prussian Blue staining solution was acquired from Leagene Biological Technology Co. Ltd. (Beijing, China). Antibodies against p53, acetylated p53, CD86, and CD206 were from Cell Signaling Technology (Danvers, MA), and IL‐1, IL‐10, TGF‐β, and TNF‐α were purchased from GeneTex (San Antonio, TX). p21, p300, CBP, p65, phosphorylated p65, and glyceraldehyde 3‐phosphate dehydrogenase (GAPDH) were purchased from Abcam (Cambridge, MA). FITC‐CD86 and APC‐CD206 were purchased from Thermo Fisher Scientific (Waltham, MA).

### 2.2 Cell culture and chemical compounds

RAW 264.7 murine macrophages and mouse hepatoma H22 cells were purchased from the American Type Culture Collection (Rockville, MD) and maintained in DMEM (HyClone, Logan, UT) with 10% FBS and 1% penicillin and streptomycin in 37°C humidified air containing 5% CO_2_. NAC (Beyotime) was dissolved in deionized water at a concentration of 8 mmol/L, and 2.5‐mg ferrous citrate or ferric citrate was dissolved in 1 mL threefold distilled water and then filtered with a 0.2‐μm filter to ensure sterility. The concentration of iron was used as previously described to avoid cytotoxicity.^12^ A p300/CBP inhibitor (Abcam) was dissolved in dimethyl sulfoxide (DMSO; Thermo Fisher Scientific) at a concentration of 25 mmol/L in up to 3 μL per 1 mL medium to prevent cytotoxicity from high concentrations of DMSO.

### 2.3 Animals and experimental protocol

Ten female BALB/c mice (6 weeks old) were housed in a pathogen‐free environment and used for H22 hepatoma cell xenografts. Five‐milligram ferric citrate at 200 μL/mouse or 200 μL saline (control) was injected intraperitoneally per 3‐day for seven times for in vivo iron loading. Xenografts were prepared by the subcutaneous injection of 1 × 10^6^ H22 cells. Tumor volume was measured every 3 days, and was calculated as previously described.^18^ The subcutaneous tumors were harvested and tumor weight was calculated on day 21 using the following equation:

$$\text{Tumor}\;\text{volume}\;=\;1/2\;(\text{length}\;\times \;{\text{width}}^{2})$$

BALB/c mice were purchased from the Experimental Animal Center of Chongqing Medical University (Chongqing, China). Animals received humane care in accordance with the guidelines provided by the National Institutes of Health for the use of animals in laboratory experiments. The animal protocols used in this work were evaluated and approved by the Animal Use and Ethic Committee of 2nd Affiliated Hospital of Chongqing Medical University (Protocol 2015‐18; Chongqing, China).

### 2.4 Western blot analysis

Macrophages were pretreated with a p300/CBP inhibitor for 1 hour. The DMSO percentage was kept at 0.3% to avoid cytotoxicity and was cultured with cells for 0‐8 hours. Cells were collected and lysed with radioimmunoprecipitation assay buffer (50 mmol/L Tris‐HCl [pH 7.4], 150 mmol/L NaCl, 1% [v/v] NP‐40, 0.1% [w/v] SDS, 0.5% [w/v] sodium deoxycholate) containing protease inhibitors and phosphatase inhibitors, and cell lysates were centrifuged at 12 000 × *g* (4°C for 10 minutes). Total protein (30 μg) was mixed with one‐quarter loading buffer (62.5 mmol/L Tris‐HCl [pH 6.8], 10% glycerol, 2% SDS, 2% β‐mercaptoethanol, and bromophenol blue), boiled for 10 minutes, and subjected to 10% or 12% SDS‐PAGE. Proteins were transferred to polyvinylidene difluoride membranes (Millipore, Bedford, MA). After the membranes were blocked in Tris‐buffered saline containing 0.05% Tween‐20 (TBST) and 5% fat‐free milk, the membrane was incubated overnight at 4°C with primary antibodies in TBST with 5% bovine serum albumin. The next day, the membranes were further incubated with the corresponding horseradish peroxidase‐conjugated secondary antibody at 37°C for 1 hour and then washed three times with TBST. Band signals were analyzed and scanned using Quantity One Software (Bio‐Rad, Hercules, CA) after incubation with an enhanced chemiluminescence reagent (Millipore).

### 2.5 RNA extraction and quantitative PCR

Total RNA was isolated from macrophages using Trizol reagent (Invitrogen) according to the manufacturer's protocol. Quantitative PCR was performed using SYBR^®^‐Green (Takara, Dalian, China) and the ABI Prism 7900 Sequence Detection System (Applied Biosystems, Foster City, CA) according to the manufacturer's protocol. The primers used were as follows: CD206 forward, 5′‐GGGACTCTGGATTGGACTCA‐3′ and reverse, 5′‐CCAGGCTCTGATGATGGACT‐3′; arginase‐1 (Arg‐1) forward, 5′‐CCCCAGTACCAACAGGACTACC‐3′ and reverse, 5′‐TGAACGTGGCGGAATTTTGT‐3′; TNF‐α forward, 5′‐GGATCTCAAAGACAACCAAC‐3′ and reverse, 5′‐ACAGAGCAATGACTCCAAAG‐3′; iNOS forward, 5′‐CTGCAGCACTTGGATCAGGAACCTG‐3′ and reverse 5′‐GGAGTAGCCTGTGTGCACCTGGAA‐3′; p53 forward, 5′‐GAGGATTCACAGTCGGATA‐3′ and reverse, 5′‐ATCATCTGGAGGAAGAAGTT‐3′; GAPDH forward,5′‐CACCCACTCCTCCACCTTTG‐3′ and reverse, 5′‐CCACCACCCTGTTGCTGTAG‐3′. Data were normalized to the expression of GAPDH.

### 2.6 Prussian staining

Macrophages were seeded in 6‐well plates and cultured in 2‐mL complete DMEM medium for 12 hours. Then, 200‐μL ferric sulfate (2.5 mg/mL), ferrous sulfate heptahydrate (2.5 mg/mL), and ferric citrate (2.5 mg/mL) were added to the medium and incubated for 12 hours. After incubation, the medium was removed and the cells were washed three times with phosphate‐buffered saline (PBS). Following fixation for 10 minutes in 4% paraformaldehyde at room temperature, the cells were incubated with Prussian Blue staining solution (1:1 mixture of 1 mol/L l‐1 hydrochloric acid and potassium ferrocyanide) for 30 minutes. Under high‐power magnification (400×), micrographs of blue‐stained cells were screened and captured using a light microscope (Leica Microsystems, Wetzlar, Germany).

### 2.7 Tissue immunofluorescence

The expression levels of CD86 in subcutaneous tumor tissues (n = 9) were measured by immunofluorescence as previously described.^18^ Cell nuclei were stained with 4′,6‐diamidino‐2‐phenylindole (DAPI). Under high‐power magnification (200×), micrographs of tissue immunofluoresence were screened and captured using a fluorescence microscope (Leica Microsystems).

### 2.8 Flow cytometry

A total of 1 × 10^6^ RAW cells were collected and washed with PBS three times following treatment with iron with or without NAC, and then fixed in 4% paraformaldehyde for 10 minutes. Cell membranes were perforated with 0.3% Triton X‐100 for 5 minutes, and FITC‐CD86 antibody (0.125 μg/test, Thermo Fisher Scientific) and APC‐CD206 antibody (0.2 μg/test, Thermo Fisher Scientific) were incubated with the macrophages for 30 minutes on ice. Finally, PBS was added to the tubes to keep the final volume at 200‐300 μL for flow cytometry (BD Pharmingen, San Diego, CA). Then, the RAW cells were collected and washed with PBS three times following iron treatment. Cells were centrifuged at 1000 × *g* for 5 minutes, after which the supernatant was discarded and 195‐μL Annexin V‐FITC binding buffer was used to gently suspend the cells. Then, 5‐μL Annexin V‐FITC and 10‐μL propidium iodide were added to the binding buffer, followed by incubation for 10‐20 minutes at room temperature in the dark.

### 2.9 ROS level detection

Macrophages were cultured with 2‐mL medium with or without NAC (8 mmol/L) for 1 hour, and then exposed to ferrous citrate or ferric citrate solution (2.5 mg/mL) for 2 hours. ROS levels in macrophages after iron treatment were measured using the 2,7‐dichlorofluorescin diacetate (DCFH‐DA) probe (Beyotime Company). Then, 1 × 10^4^ macrophages were culture in 96‐well plates for 4 hours and then treated with iron for 2 hours, after which cells were washed three times with PBS before incubation in 200‐μL serum‐free medium with DCFH‐DA (1000:1) for 20 minutes at 37°C. Then, cells were washed three times with serum‐free medium, and fluorescence was measured at 485‐nm excitation and 520‐nm emission using a microplate reader (Promega, Fitchburg, WI). The values are presented as fluorescence intensity relative to the controls. The cells were observed with a fluorescence microscope (Leica Microsystems), and the images were captured at a 200× magnification.

### 2.10 Statistical analysis

All data were analyzed with SPSS 17.0 software (SPSS Inc., Chicago, IL). The results are presented as mean ± standard deviation. Comparisons among groups were performed using one‐way analysis of variance. The statistical differences between groups were determined by the Student's *t* test. *P* values less than 0.05 were considered statistically significant.

## 3 RESULTS

### 3.1 Iron overload induced M1 macrophage polarization

**Figure 1:**
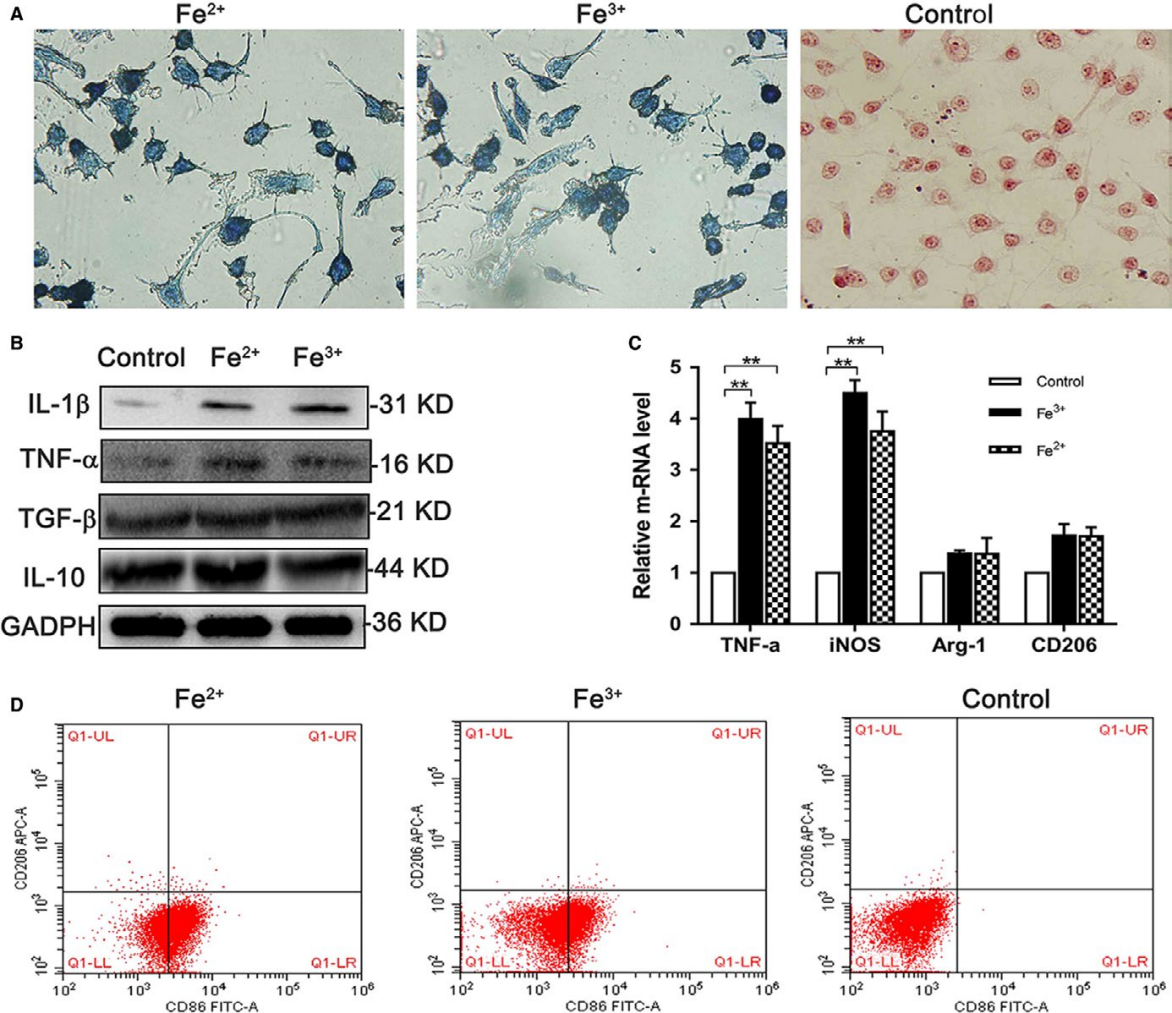
Iron‐induced macrophages polarized to M1 subtype. A, Prussian blue staining showed that macrophages rapidly take up iron ion (magnification ×400). B‐D, Western blotting showed that M1 macrophages–associated markers IL‐1β and TNF‐α were induced by iron, and M2 macrophages–associated markers IL‐10, TGF‐β did not change significantly. The results of qRT‐PCR showed that TNF‐α and iNOS, the markers of M1 macrophage, were highly expressed (*P* < 0.05), and the M2 macrophages–associated markers Arg‐1 and CD206 expression level did not change significantly (*P* > 0.05). The results of FCM showed that macrophages highly expressed CD86 after iron treatment, and CD206 did not change significantly. **P < 0.05

RAW 264.7 cells were treated with 200‐μL filter‐sterilized ferric citrate (2.5 mg/mL), ferrous citrate (2.5 mg/mL), or PBS (control) for 2 hours. The results of Prussian staining are shown in Figure 1A, and showed that macrophages could rapidly take up iron ion. Considering that previous studies have shown a relationship between intracellular iron concentration and macrophage polarization,^3^, ^5^ we measured the expression of IL‐1β, IL‐10, TNF‐α, and TGF‐β with Western blotting (Figure 1B), the result showed expression of IL‐1β and TNF‐α increased. The results of qRT‐PCR and flow cytometry are shown in Figure 1C,D, and showed that both Fe^2+^ and Fe^3+^ induced the expression of M1‐associated maker such as TNF‐α, CD86, and iNOS. Considering that ferric citrate and ferrous citrate had the same effects which polarized macrophages to M1 subtype, only ferric citrate was used as a stimulator in our subsequent experiments. Fe^2+^ and Fe^3+^ probably had the same polarization effects because of Fenton's reaction.

### 3.2 Iron polarized macrophages to the M1 phenotype by inducing intracellular ROS production

**Figure 2:**
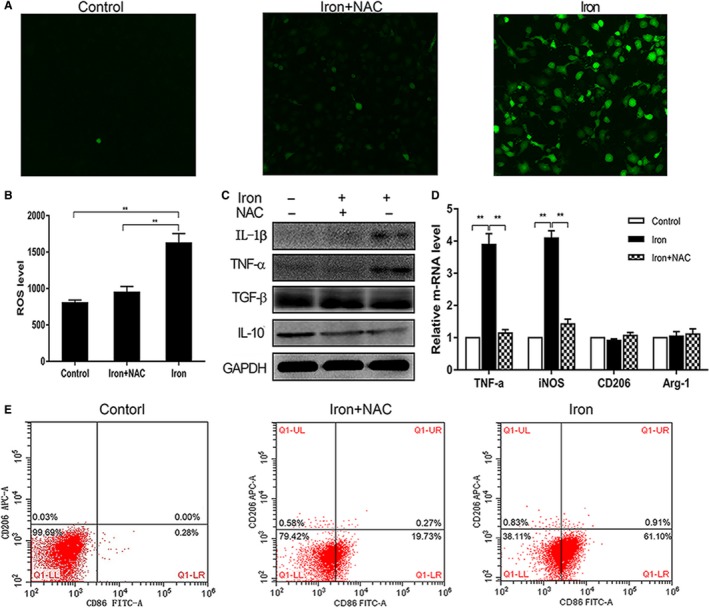
Iron‐polarized macrophages to M1 phenotype by inducing intracellular reactive oxygen species (ROS) production. A, B, DCFH‐DA probe was used to detected ROS with fluorescent microscopy and microplate reader. The intensity of green fluorescence and the values were correlated with ROS level (magnification ×400). And *N*‐acetyl‐l‐cysteine (NAC) could effectively inhibit the production of ROS which induced by iron. C‐E, Results of Western blotting, qRT‐PCR and FCM showed NAC inhibited the expression of CD86, IL‐1β, TNF‐α and iNOS (*P *<* *0.01), which induced by iron over‐loaded, and TGF‐β, IL‐10, CD206, and Arg‐1 did not change significantly (*P *>* *0.05). *P <* *0.05, **P <* *0.01

RAW 264.7 cells were treated with 200‐μL filter‐sterilized ferric citrate (2.5 mg/mL) or 200‐μL PBS for 2 hours with or without a 1‐hour pretreatment with NAC (8 mmol/L), and DCFH‐DA was used to detect intracellular ROS with a fluorescence microscope and microplate reader as shown in Figure 2A,B. Iron induced ROS production rapidly, which could be repressed by NAC (Figure 2A,B). The results of flow cytometry, Western blotting, and qRT‐PCR showed that iron polarized macrophages toward the M1 subtype, which was inhibited by NAC (Figure 2C‐E). These data suggested that iron overload induced proinflammatory polarization through ROS production.

### 3.3 ROS production induced p53 expression

**Figure 3:**
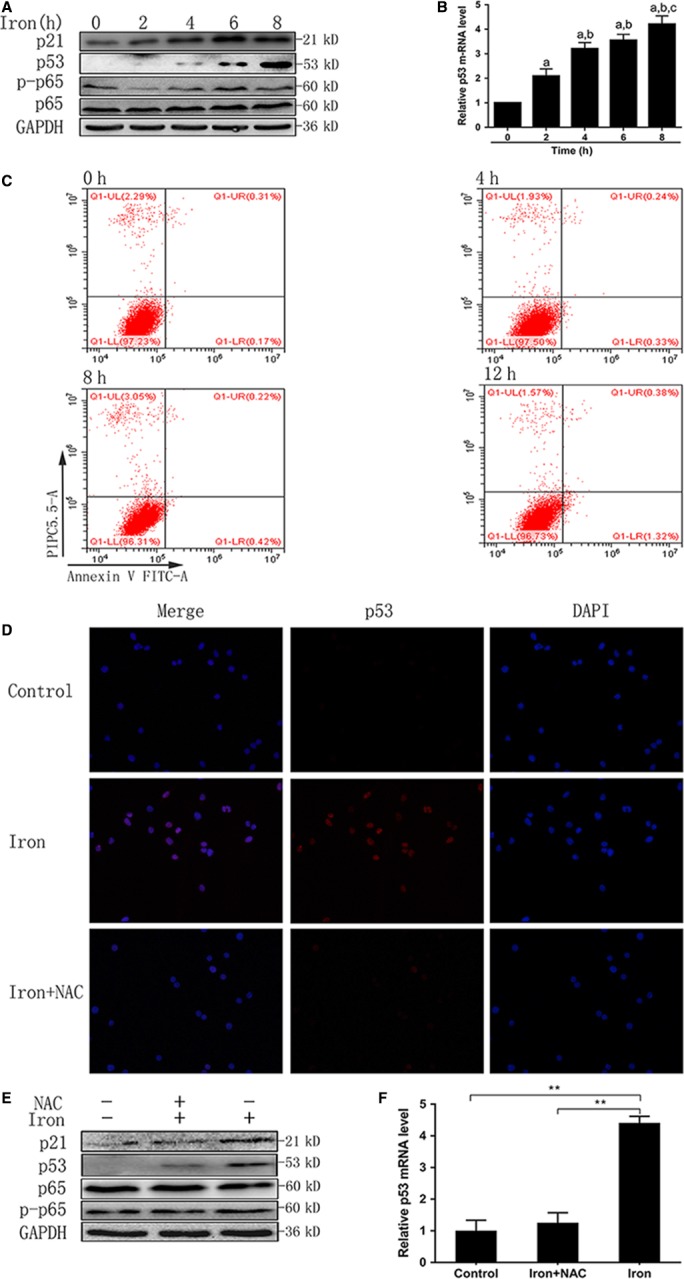
Reactive oxygen species production–induced p53 expression. A, B, Western blotting and qRT‐PCR showed that iron induced the expression of p53 and p21 in a time‐dependent manner (*P *<* *0.01). C, Flow cytometry showed that iron treatment did not increase the apoptotic rate of RAW cells (*P *>* *0.05). D, Immunofluorescent staining showed that *N*‐acetyl‐l‐cysteine (NAC) inhibited the expression of p53 (red), which induced by iron treatment (magnification ×400). E, F, Western blotting and qRT‐PCR showed that NAC repressed p53 expression (*P *<* *0.01). a: *P *< 0.01 vs 0 h, b: *P* < 0.01 vs 2 h, and c: *P* < 0.01 vs 4 h; **P <* *0.01

P53 plays a key role in cell oxidative stress, DNA repair, and iron metabolism.^19^ Thus, we speculated that p53 might be involved in M1 polarization induced by ROS. RAW264.7 macrophages were treated with filter‐sterilized ferric citrate (2.5 mg/mL) for 0‐12 hours. We evaluated the expression of p53, p21, p65, and phosphorylated p65 in macrophages after iron treatment, and found that iron induced the expression of p53 and its downstream protein p21 in a time‐dependent manner without increasing the expression of total or phosphorylated p65 expression (Figure 3A,B). Flow cytometry showed that iron treatment did not increase the apoptotic rate of RAW cells (Figure 3C). RAW 264.7 macrophages were pretreated with NAC (8 mmol/L) for 1 hour to repress the production of ROS, and then treated with iron (2.5 mg/mL) for 4 hours. Reduction in ROS levels by NAC repressed p53 expression (Figure 3D‐F). These data suggested that ROS increased the expression of p53, which might play a role in iron overload‐induced M1 polarization.

### 3.4 ROS production promoted M1 polarization by inducing p53 acetylation

**Figure 4:**
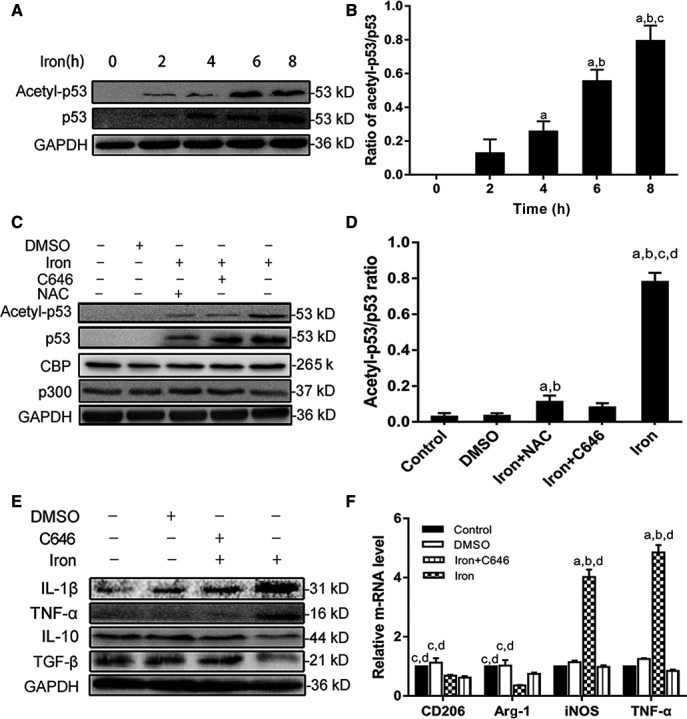
Reactive oxygen species–promoted M1 polarization through inducing acetylation of p53. A, B, RAW cells were treated with iron (2.5 mg/mL) for 0, 2, 4, 6, and 8 h. C, D, Macrophages were pretreated for 1 h c646 (25 mmol/L) or *N*‐acetyl‐l‐cysteine (NAC) (8 mmol/L), followed by 8‐h iron treatment (2.5 mg/mL). E, F, Macrophages were divided into five groups: control group, DMSO (0.3%) group, NAC + iron group (NAC group), C646 + iron group (C646 group), and iron group. And control group was pretreated with PBS, DMSO group was pretreated with DMSO (0.3%), NAC group was pretreated with NAC (8 mmol/L), and C646 group was pretreated with p300/CBP inhibitor (25 mmol/L) for 1 h. Then all group received an 8‐h iron treatment (2.5 mg/mL). A, B, Western blotting showed that p53 acetylation increased in a time‐dependent manner (*P *<* *0.05). a: *P *< 0.05 vs 2 h, b: *P* < 0.01 vs 4 h, and c: *P* < 0.01 vs 6 h. C, D, Western blotting showed that NAC treatment reduced acetyl‐p53 (*P *< 0.01) and p53 compared with iron group. And C646 inhibited the acetylation of p53 compared with iron group (*P* < 0.01). The expression of p300/CBP did not change in all group. a: *P *< 0.05 vs control, b: *P* < 0.05 vs DMSO, c: *P* < 0.01 vs iron + NAC, and d: *P* < 0.01 vs iron + C646. E, F, Western blotting and qRT‐PCR showed iron treatment polarized macrophages to M1 phenotype (*P * < * *0.01), and C646 abolished the effect of M1 polarization induced by iron treatment (*P * < * *0.01). a: *P* < 0.01 vs control, b: *P* < 0.01 vs DMSO, c: *P* < 0.01 vs iron, and d: *P* < 0.01 vs iron + C646

We found that acetyl‐p53 in macrophages increased as p53 expression increased after iron treatment (Figure 4A,B). P53 acetylation was correlated with p53 activation during the DNA damage response. To determine the effects of p53 acetylation in M1 polarization, we inhibited the p53 acetylase in macrophages with the p300/CBP inhibitor C646 as Zheng described.^20^ Figure 4C,D showed that C646 repressed p53 acetylation but did not decrease total p53 level after iron treatment, whereas NAC repressed p53 expression and acetylation. C646 reversibly inhibited the activity of p300 and CBP acetyltransferase, but did not affect the expression of p300 and CBP acetyltransferase as Erin M. Bowers described.^21^ Western blotting and qPCR were used to detect the polarization index. As shown in Figure 4E,F, IL‐1β and TNF‐β were reduced by C646, but expression of IL‐10 and TGF‐β was unaffected, suggesting that iron polarized macrophages to the M1 subtype and C646 erased the effects on M1 polarization of iron oversload. These results suggested that ROS production induced by iron overload enhanced the activity of p300/CBP acetyltransferase, thereby increasing the acetylation of p53 and promoting M1 polarization.

### 3.5 Iron polarized macrophages to the M1 phenotype in BALB/c mice

**Figure 5:**
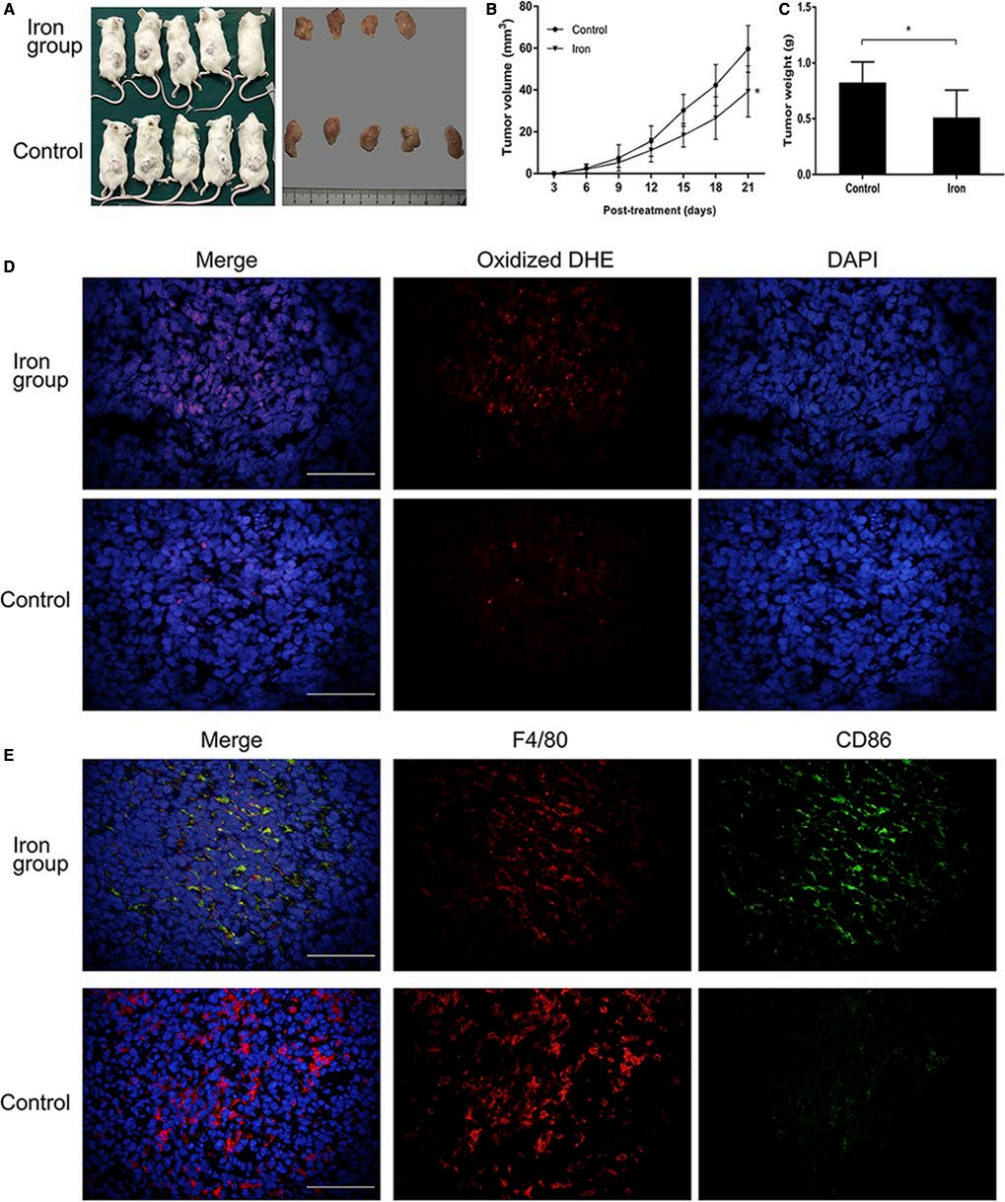
Iron‐polarized macrophages to M1 subtype in subcutaneous tumor of BALB/c mice. A, B, The volumes of tumor in the iron‐treated group (n=4) were significantly smaller than those in the control group (n = 5), *P <* *0.05 vs control at day 21. C, Mean weight of tumors in the iron‐treated group (n=4) was significantly lighter than that in control group (n = 5), *P <* *0.05 vs control. D, Oxidized DHE (red) located in the nucleus, and iron‐treated group contained more oxidized DHE than control group (magnification ×400). E, CD86 expression in the iron‐treated group was more than that in the control group (magnification ×400)

We confirmed that iron overload induced M1 polarization in vitro. To determine if higher serum level of iron could enhance the antitumor activity of macrophages in mice, 200‐μL filter‐sterilized ferric citrate solution (2.5 mg/mL) or saline (control) was tail intravenous injected into BALB/c mice per 3‐day on day 1. H22 hepatoma cells were subcutaneously injected into mice on day 1, and tumor growth was measured every 3 days, and the mice were sacrificed on day 21. Iron‐treated mice had smaller subcutaneous tumors than the control group (Figure 5A‐C). To detect ROS production in the subcutaneous tumors, frozen sections were stained with DHE. Iron‐treated mice had increased production of ROS, as detected using DHE, a marker of oxidative stress (Figure 5D). To determine if iron overload had a polarization effect on subcutaneous macrophages, we detected M1 macrophages with CD86 antibody and total macrophages with F4/80 antibody using immunofluorescence staining. Figure 5E showed that macrophages in the subcutaneous tumors of iron‐treated mice were obviously polarized to the M1 subtype, but macrophages in subcutaneous tumors of the control group barely expressed CD86. These results indicated that macrophages in the subcutaneous tumors were induced to the M1 phenotype by high serum level of iron.

## 4 DISCUSSION

Liver and macrophages play important roles in iron metabolism,^1^ but the changes on polarization of macrophages during the process of iron accumulation are not fully understood, and the relationship between iron overload and macrophage polarization has been the subject of debate. During our research study, we found that iron overload promoted macrophage polarization to the M1 phenotype concomitant with ROS production. Intracellular free iron ions can convert O2^• −^ and/or H_2_O_2_ into OH^•^ through Fenton Chemistry (Fe^2+^ + H_2_O_2_ → Fe^3+^ + OH^−^ + OH^•^). Considering that p53 plays an important role in regulating the metabolism of iron and the polarization of macrophages^19^, ^22^ and is also a protective factor for various stimuli such as ROS, we measured the expression of p53 by Western blotting and qRT‐PCR. We found that expression of p53 and its downstream target p21 increased in macrophages after iron treatment without an increase in apoptosis, and the NF‐κB pathway‐associated protein p65 and phosphorylated p65 did not significantly change. The expression of p53 and p21 was inhibited by NAC through decreasing the production of ROS. The reduction ni ROS by NAC inhibited expression of the M1 polarization index. These results showed that p53 might be involved in M1 polarization induced by ROS production.

The acetylation of p53 is mediated by p300 and CBP acetyltransferases, which strongly potentiate p53‐dependent transcriptional activation. Acetylation of p53 inhibits nonspecific DNA binding, enhances sequence‐specific DNA binding activity, is a protective factor for DNA damage, and causes either cell cycle arrest and DNA repair or apoptosis.^23^, ^24^ Thus, we speculated that ROS not only induces p53 expression but also activates p53 to repair DNA damage. We found that acetyl‐p53 was increased in a time‐dependent manner after iron treatment, suggesting that p53 acetylation might play a role in macrophage polarization. To test the role of acetylated p53 in M1 polarization, we used C646 to inhibit p53 acetylation in macrophages. Previous studies have shown that C646 attenuates p300 acetyltransferase activity and inhibits p53 acetylation.^21^ We found that iron treatment did not polarize macrophages to the M1 subtype following C646 treatment. In addition, NAC treatment also inhibited M1 polarization by reducing ROS production, whereas C646 inhibited M1 polarization by decreasing p53 acetylation without reduction in ROS production. These results indicate that high ROS levels induced by iron treatment increased the activity of p300/CBP acetyltransferase and promoted p53 acetylation, leading to M1 macrophage polarization. In consideration of that C646 caused the suppression of acetylation in p53, MyoD, and NF‐κB,^25^ a p53 acetylation inhibition assay is required to provide more evidence.

To determine if higher serum levels of iron could enhance macrophage antitumor activity in mice, H22 cells were subcutaneously injected into 10 BABL/c mice with or without a pretreatment of iron. The volume and weight of subcutaneous tumors exhibited significant differences between the iron‐treated group and control group. In addition, the subcutaneous tumors of the iron‐treated group produced more ROS than the control group. Immunofluorescence staining showed that M1 polarization was more obvious in the iron‐treated group than control group. All results indicated that iron overload in macrophages induced ROS production and enhanced the activity of p300/CBP acetyltransferase, thus increasing p53 acetylation and polarizing macrophages to the M1 subtype, thus enhancing the antitumor effect of tumor‐infiltrating macrophages.

## 5 CONCLUSIONS

The results of this study showed that p53 acetylation induced by ROS played an important role in M1 polarization induced by iron accumulation in macrophages, and iron accumulation polarized macrophages to the M1 phenotype. Appropriately increasing the level of iron in cancer patients and promoting the polarization of tumor‐associated macrophages to the M1 type may improve antitumor immunity in cancer patients. However, how to reduce the side effects caused by high levels of iron, such as endothelial cell damage and liver cirrhosis, requires further studies.

## CONFLICT OF INTEREST

The authors declare that they have no conflict of interest.
